# Regional economic development, household income, gender and hypertension: evidence from half a million Chinese

**DOI:** 10.1186/s12889-020-09002-y

**Published:** 2020-06-10

**Authors:** Kun Tang, Yu Zhang, Hanyu Wang, Shi Hui Tan, Lin Bai, Yuning Liu

**Affiliations:** 1 Vanke School of Public Health, Tsinghua University, Beijing, China; 2 School of Health Humanities, Peking University Health Science Center, Beijing, China; 3 School of Basic Medical Sciences, Peking University Health Science Center, Beijing, China; 4 School of Pharmaceutical Sciences, Peking University Health Science Center, Beijing, China; 5 School of Public Health, Peking University Health Science Center, Beijing, China

**Keywords:** Regional economic development, Prevalence of hypertension, Gender differences, Household income, China

## Abstract

**Background:**

Hypertension may be influenced by multiple factors, including social and individual determinants. Regional and individual economic disparity in China is closely associated with such factors that may give rise to diverse health outcomes. This study examines the relationship between regional economic development, household income, gender and hypertension prevalence in China.

**Methods:**

This study utilized data from the China Kadoorie Biobank (CKB), a population-based study on half a million Chinese adults from 10 geographically distinct regions. Hypertension was identified by a measured systolic blood pressure/diastolic blood pressure ≥ 140/90 mmHg or receiving treatment. Regional economic development was inferred from GDP per capita at the time of the study. A logistic regression based method was used in calculating the prevalence of hypertension in different household income, regional economic development, and gender groups, adjusting for demographic, social-economic and lifestyle factors.

**Results:**

The prevalence of hypertension was the lowest in the medium GDP per capita areas in both male (31.62, 95% CI: 31.26–31.98%) and female (22.85, 95% CI: 22.50–23.19%) as compared to that in the low GDP per capita regions (male: 32.75, 95% CI 32.41–33.08%; female: 32.12, 95% CI: 31.78–32.47%) and high GDP per capita areas (male: 39.74, 95% CI: 39.33–40.16%; female: 35.19, 95% CI: 34.74–35.65%). There was an inverse relationship between hypertension and household income in the low and high GDP areas and an U-shaped association in the medium GDP per capita areas. Higher hypertension prevalence was observed in males across all GDP per capita areas. The negative correlation between hypertension and household income (across all GDP per captia areas) was stronger in females than in males.

**Conclusions:**

The present study underlined the important influence of regional economic development, household income and gender on hypertension. Interventions for hypertension prevention and management should take into consideration the influence of sex difference and socioeconomic disparities at both micro- and macro- levels, in addition to a person-centered approach.

## Background

Hypertension is one of the leading risk factors for cardiovascular diseases and chronic kidney diseases [1]. The number of adults with hypertension increased from 594 million in 1975 to 1.13 billion in 2015, occurring mostly in the low- and middle-income countries [2]. According to a recent national cohort study, one-third of Chinese adults have hypertension, which brings about significant socioeconomic burdens [3]. As a global approach, the World Health Assembly in 2013 adopted the global non-communicable disease targets, one of which was to lower the prevalence of raised blood pressure [4].

China has undergone rapid urbanization in the past three decades [5], which brings regional disparity in the economy and social development [6]. These societal and economic changes have brought about health-related changes, including environment, dietary habits, physical activities and healthcare systems [7–9]. Income disparity and social inequality are negatively related to health outcomes [10].

The correlations between regional economic conditions, income and prevalence of hypertension are complex. Better economic condition is generally considered to be beneficial to health [11], while income inequalities accompanied by economic development in a region is observed to have negative effects on health [12]. Socioeconomic status is found to have a negative association with hypertension in the developed countries [13] and a positive association in the developing countries. To better illustrate the interplay between socioeconomic status, regional development and hypertension prevalence, a conceptual framework was added as Additional file 1*.*

To our knowledge, this is the first study to explore the relationship between income, regional economic development, and prevalence of hypertension in China. This paper aimed to investigate whether regional economic status can influence hypertension prevalence and also influence the association between household income and hypertension with consideration of gender difference.

## Methods

### Sample

We used data from the CKB study, a population-based research in adults aged 30–79 from 10 geographically defined areas in China from 2004 to 2008 [14, 15]. The 10 regions were selected according to local disease patterns and risk exposures, population stability, quality of death and disease registries, and economic development. The selection provided approximately equal coverage of rural (Gansu, Henan, Sichuan, Hunan, and Zhejiang) and urban (Harbin, Qingdao, Suzhou, Liuzhou, and Haikou) provinces, in which approximately 44.6% of participants were from urban regions. In each region, permanent residents who were physically able to participate were invited to participate. Potential participants were approached in person by community leaders or health workers. The estimated population response rate was about 30% (26–38% in the five rural areas and 16–50% in the five urban areas) [15]. A total of 512,891 individuals (representing approximately 30% of the total population of the 10 regions sampled) completed the interviewer-administered computerized questionnaire and clinic visits. A series of physical measurements were completed during clinic visits, including height and weight, hip and waist circumference, bio-impedance, systolic and diastolic blood pressure (mmHg) and lung function.

### Exposures

In the present study, annual household income was characterized by a five-category variable: no income or ≤ 4999 Chinese Yuan (1 USD ≈ 7.61 Chinese Yuan in 2007); 5000–9999 Yuan; 10,000–19,999 Yuan; 20,000–34,999 Yuan; and ≥ 35,000 Yuan. We combined the first two categories (no income or ≤ 2499 Yuan; 2500–4999 Yuan) in order to balance the requirement for the minimum sample size in each category and also to allow for comparisons among various transitional groups from lowest to highest income. The midpoint of each category (starting point of the last category) was used to represent the average income.

We collected GDP per capita in the year 2007 from the statistical yearbooks of local government. The lowest GDP per capita in the sample was 5550 Yuan (Maijixiang, Tianshui, Gansu), while the highest was 69,151 Yuan (Suzhou, Jiangsu). Detailed GDP per capita in the 10 study regions can be found in Additional file 1 for the geographic distribution of the study areas. Regional economic development was classified into three levels: low GDP per capita areas: (5000 Yuan–19,999 Yuan); medium GDP per capita areas: (20,000 Yuan–29,999 Yuan); and high GDP per capita areas: (30,000 Yuan and above). The criteria for categorization was to balance the number of regions and population in each category.

### Outcome

Blood pressure was measured twice by trained staff using a digital sphygmomanometer (Omron UA-779) after participants had remained at rest in the seated position for at least 5 min. In case the difference between the two measurements was greater than 10 mmHg for systolic blood pressure, a third measurement was obtained and the mean of the last two measurements was used for analysis. All devices were regularly maintained and calibrated to ensure the consistency of the measurements. Further details of the CKB study were described in the previous publication [15]. In this study, we used the 1999 WHO/ISH (World Health Organization/ the International Society of Hypertension) guidelines as reference in categorizing hypertensive participants [16]. Those with a measured systolic blood pressure ≥ 140 mmHg or a measured diastolic blood pressure ≥ 90 mmHg or were receiving treatment for hypertension were considered as hypertensive [16]. Receiving treatment for hypertension was defined as those who reported a diagnosis of hypertension by a physician and the use of antihypertensive medications at baseline.

### Other covariates

Demographic and socioeconomic characteristics, including age, gender, highest level of education, and occupation, were collected from the baseline survey. In order to balance the group size, we recategorized age, education and occupation from the original questionnaire. Age was categorized into two groups: < 55 years and ≥ 55 years old. Highest level of education was categorized into three groups: uneducated and primary school, middle and high school, college/university graduate and above. Occupation was categorized into agriculture and related workers, factory workers, clerks (i.e. administrator/manager, professional/technical, sales and service workers, self-employed and others), and unemployed (i.e. unemployed, retired and house wife/husband). Total physical activity was calculated as metabolic equivalent task hours (MET-hours/day) spent on work, transportation, housework, and non-sedentary recreation and sedentary leisure time was quantified as hours per day. MET values were categorized into four groups: < 11.0 h/d, 11.0–19.0 h/d for men or 11.0–17.0 h/d for women, 19.0–32.5 h/d for men or 18.0–28.5 h/d for women, > 32.5 h/d for men or > 28.5 h/d for women. Smoking habits and alcohol use were self-reported and were classified as “frequent,” “occasional,” and “non” smoker/drinker. Trained workers measured weight, height, waist and hip circumference using calibrated instruments. Body Mass Index (BMI) was calculated as weight in kilograms divided by height in meters squared [15]. BMI (kg/m^2^) was categorized as < 18.5, 18.5–23.9, 24.0–27.9, and ≥ 28.0 kg/m^2^, based on the standard classification specific for the Chinese population [17]. Sleep duration, depression and anxiety status were included as mental health factors. Sleep duration was categorized into three groups:≤6 h, 6-9 h, > 9 h. Depression and anxiety conditions were accessed by trained health workers at the study clinic using the Chinese version of the computerized Composite International Diagnostic Inventory — short form (CIDI-SF) [18] .

### Data analysis

The basic demographic, socioeconomic, lifestyle factors in low, medium, and high GDP regions were illustrated using descriptive analyses. A logistic-regression-based method was used to calculate the adjusted prevalence of hypertension, anxiety and depression. Detailed method was described elsewhere [19, 20]. Briefly, this method is based on logistic regression. Adjusted values were calculated using the floating method. By attributing variance to the reference group, the floating method allows for comparisons of risks between any two groups [21]. Prevalence of hypertension in males and females living in different GDP per capita areas was calculated, adjusted for age, occupation, education, MET, BMI, alcohol, smoke, sleeping time, anxiety, depression, household income and household size. Prevalence of hypertension in both sexes with different household income was calculated, adjusted for age, occupation, education, MET, BMI, alcohol, smoke, sleeping time, anxiety, depression, regional economic development and household size. Smooth curves were plotted with standard Microsoft Excel smoothing (based on a Catmull-Rom spline) to demonstrate the trend of hypertension prevalence in different GDP per capita areas with changing household income stratified by gender. Prevalence of hypertension was also calculated for other socioeconomic, lifestyle and mental health categories adjusting for covariates abovementioned and stratified by regional GDP per capita level and gender. Covariates were selected based on significances obtained in the univariate analyses (presented in Additional file 1) and results from relevant literature [3, 22–25]. Analyses were conducted using SAS 9.4 statistical software (SAS Institute, Cary NC).

## Results

### Socioeconomic, lifestyle and mental health characteristics

Basic characteristics of the study population by levels of GDP per capita are shown in Table 1. Of all 512,891 participants, more than a third lived in low GDP per capita areas (44.65%). The mean ages of the study were the highest for the population living medium GDP per capita areas (53.15) than those in low (50.53) and high GDP per capita areas (51.57). Educational attainment of most people in the low (61.96%) and high (59.90%) GDP per capita areas were uneducated and primary school, while most people had middle and high school education (61.16%) in the medium GDP per capita areas. The most common occupation in the low GDP per capita areas was agriculture and related (77.00%), and unemployment was more prevailing in the medium GDP per capita areas (56.94%).

**Table 1:** Basic characteristics of participants

	Level of economic development (measured by regional GDP per capita)							
	Low		Medium		High		Total	
	N	%	N	%	N	%	N	%
	229,001	44.65	137,417	26.79	146,473	28.56	512,891	100.00
Demographic characteristics								
Mean age, years (SD)	50.53(10.60)		53.15(11.12)		51.57(10.17)		51.53(10.68)	
< 55 years	148,186	64.71	77,922	56.70	91,992	62.80	318,100	62.02
≥ 55 years	80,815	35.29	59,495	43.30	54,481	37.20	194,791	37.98
Female, %	134,125	58.57	84,049	61.16	84,458	57.66	302,632	59.01
Socioeconomic characteristics								
Highest education, %								
Uneducated and primary school	141,886	61.96	30,811	22.42	87,740	59.90	260,437	50.78
Middle and high school	84,130	36.74	84,050	61.16	54,260	37.04	222,440	43.37
College and university	2985	1.30	22,556	16.41	4473	3.05	30,014	5.85
Household income, %								
≤ 4999 yuan	40,610	17.73	4557	3.32	5036	3.44	50,203	9.79
5000–9999 yuan	69,529	30.36	16,935	12.32	8165	5.57	94,629	18.45
10,000–19,999 yuan	74,675	32.61	47,038	34.23	27,300	18.64	149,013	29.05
20,000–34,999 yuan	30,886	13.49	39,785	28.95	56,050	38.27	126,721	24.71
≥ 35,000 yuan	13,301	5.81	29,102	21.18	49,922	34.08	92,325	18.00
Occupation, %								
Agriculture and related	176,325	77.00	4671	3.40	33,011	22.54	214,007	41.73
Factory workers	4767	2.08	21,538	15.67	46,090	31.47	72,395	14.12
Clerk	12,989	5.67	32,964	23.99	28,487	19.45	74,440	14.51
Unemployed	34,920	15.25	78,244	56.94	38,885	26.55	152,049	29.65
Lifestyle factors								
Physical activity (MET, h/d),%								
< 11.0	59,094	25.81	51,923	37.78	27,692	18.91	138,709	27.04
11.0-	58,426	23.03	45,000	29.82	30,659	18.57	134,085	26.14
Men 19.0-,Women 17.0	59,360	26.69	25,952	20.67	36,611	25.53	121,923	23.77
Men 32.5-,Women 28.5-	52,121	24.48	14,542	11.72	51,511	36.99	118,174	23.04
BMI (kg/m2), %								
< 18.5	12,023	5.25	5235	3.81	5117	3.49	22,375	4.36
18.5-	130,238	56.89	64,714	47.09	71,056	48.51	266,008	51.86
24.0-	66,989	29.25	51,049	37.15	52,131	35.59	170,169	33.18
28.0-	19,751	8.62	16,419	11.95	18,169	12.4	54,339	10.59
Smoking, %								
Never	136,728	59.71	90,750	66.04	90,136	61.54	317,614	61.93
Occasional	25,503	11.14	18,508	13.47	15,711	10.73	59,722	11.64
Regular	66,770	29.16	28,159	20.49	40,626	27.74	135,555	26.43
Alcohol, %								
Never	100,843	44.04	53,073	38.62	81,283	55.49	235,199	45.86
Occasional	91,525	39.97	57,404	41.77	35,218	24.04	184,147	35.90
Regular	36,633	16.00	26,940	19.6	29,972	20.46	93,545	18.24
Sleep duration/day,%								
≤ 6 h	50,649	22.12	39,051	28.42	28,750	19.63	118,450	23.09
> 6 h, ≤9 h	161,716	70.62	95,246	69.31	111,210	75.93	368,172	71.78
> 9 h	16,636	7.26	3120	2.27	6513	4.45	26,269	5.12
Depression Prevalence,%	1695	0.74	426	0.31	1084	0.74	3205	0.62
Anxiety Prevalence,%	595	0.26	110	0.08	249	0.17	954	0.19
Hypertension Prevalence, %	74,105	32.36	35,481	25.82	54,107	36.94	163,693	31.92
Controlled	4467	6.12	6192	14.44	5539	9.44	16,198	9.28
Treated but uncontrolled	11,566	15.86	10,075	23.50	12,264	20.91	33,905	19.43
Diagnosed but untreated	4259	5.84	2911	6.79	2430	4.14	9600	5.50
Undiagnosed	52,648	72.18	23,700	55.27	38,430	65.51	114,778	65.78
Cardiovascular Disease History	4496	1.96	8108	5.90	2868	1.96	15,472	3.02

### Prevalence of hypertension among people with different household income

**Fig. 1:**
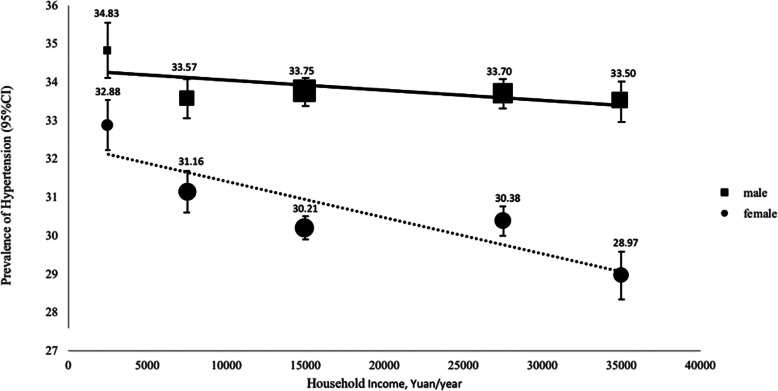
Adjusted Prevalence (95%CI)* of hypertension across different levels of household income by sex. Note: The size of square/circle in figure 1 represents the population size. * Adjusted for age at the time of study, occupation, education, BMI, METs, tabacco and alcohol use, sleep duration, depression, anxiety,, regional economic development and household size

Figure 1 presents the adjusted prevalence of hypertension by different household income. Negative associations between household income and hypertension prevalence were observed in both sexes. In males there was a slight decrease in hypertension prevalence from 34.83% (95% CI: 34.10–35.56%) in household income of ≤4999 to 33.50% (95% CI: 32.97–34.08%) in household income of ≥35,000. In females, the decrease was more obvious from 32.88% (95% CI: 32.22–33.53%) in household income of ≤4999 to 28.97% (95% CI: 28.34–29.59%) in household income of ≥35,000.

### Prevalence of hypertension among different GDP per capita areas

**Fig. 2:**
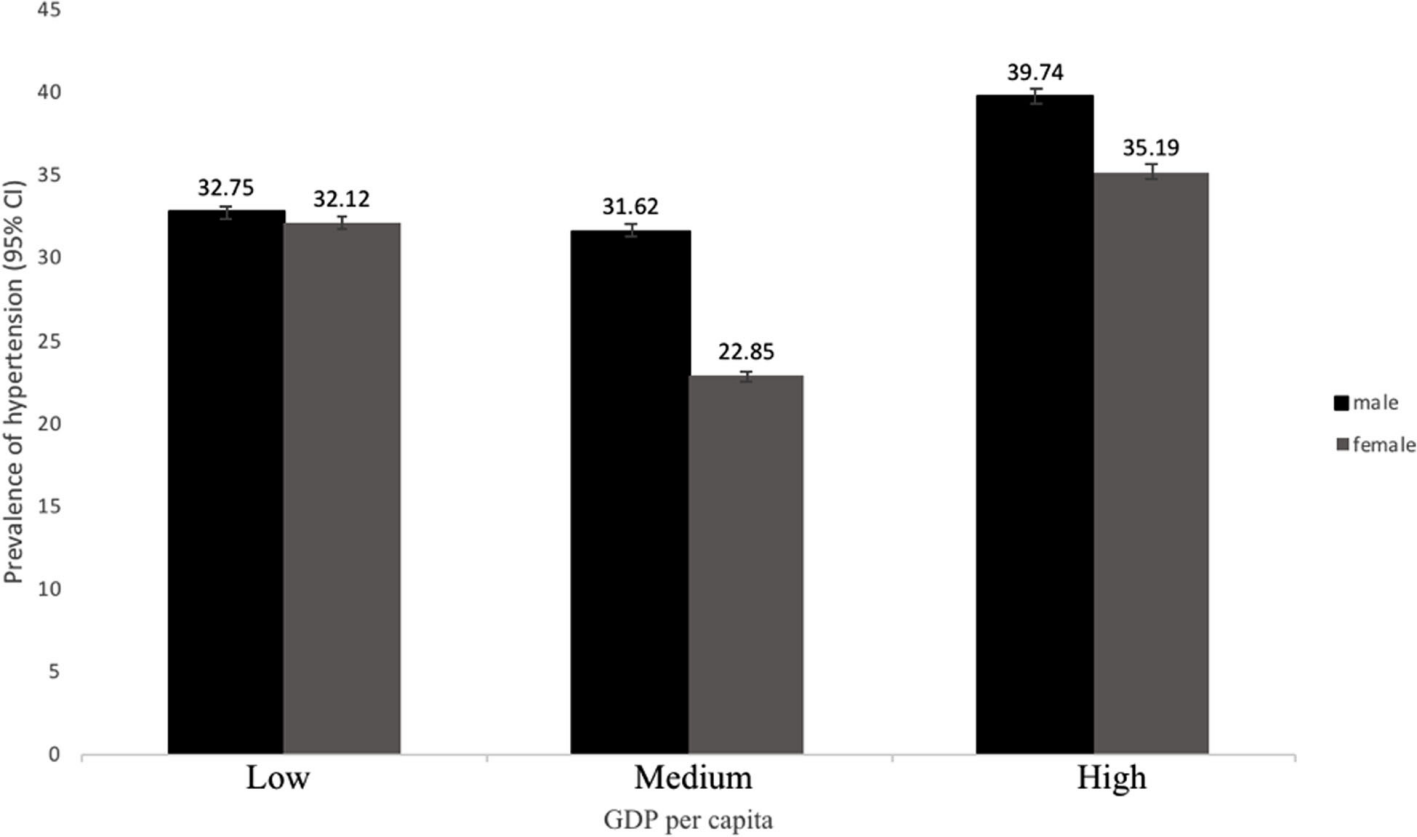
Adjusted prevalence of hypertension (95%CI)* by regional GDP per capita levels and gender. * Adjusted for age at the time of study, occupation, education, BMI, METs, tobacco and alcohol use, sleep duration, anxiety, depression, household income and household size

Figure 2 presents the adjusted prevalence of hypertension in both sexes in different GDP per capita areas. The prevalence of hypertension was the lowest in medium GDP per capita areas, and the highest in high GDP per capita areas. In males, the prevalence of hypertension was 32.75% (95% CI: 32.41–33.08%) in the low GDP per capita areas, 31.62% (95% CI: 31.26–31.98%) in the medium GDP per capita areas, and 39.74% (95% CI: 39.33–40.16%) in the high GDP per capita areas. In females, the prevalence of hypertension was 32.12% (95% CI: 31.78–32.47%) in the low GDP per capita areas, 22.85% (95% CI: 22.50–23.19%) in the medium GDP per capita areas, and 35.19% (95% CI: 34.74–35.65%) in the high GDP per capita areas. The prevalence of hypertension was also higher in males than in females across all three GDP per capita areas. Similar association was found for adjusted mean systolic and diastolic blood pressures (see Additional file 1).

### Prevalence of hypertension and income across different GDP per capita areas

**Fig. 3:**
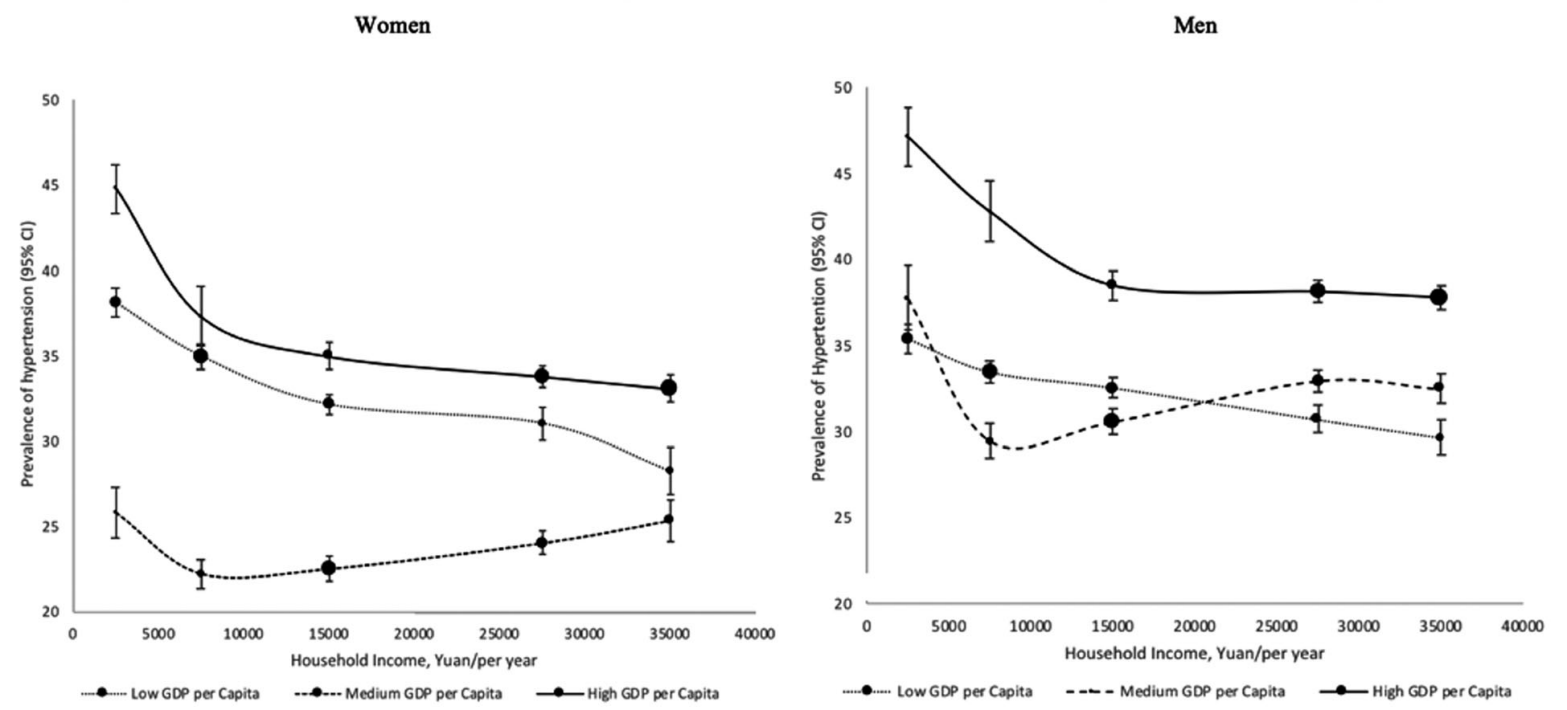
Adjusted prevalence (95%CI)* of hypertension and economic environment (regional GDP per capita and household income) in both sexes. *Adjusted for age at the time of study, occupation, education, BMI, MET, alcohol, smoke, sleeping time, anxiety, depression and household size

Figure 3 presents the adjusted prevalence of hypertension in both sexes by household income in different GDP per capita areas. There was no obvious sex difference in the shapes of association between hypertension prevalence and household income in different GDP per capita areas. For males from high GDP per capita regions, the prevalence of hypertension decreased from 47.20% (95% CI: 45.49–48.91%) in household income of ≤4999 Yuan to 37.81% (95% CI: 37.09–38.54%) in household income of ≥35,000 Yuan. The hypertension prevalence in the low GDP per capita regions decreased from 35.38% (95% CI: 34.52–36.24%) in household income of ≤4999 to 29.68% (95% CI: 28.70–30.66%) in household income of ≥35,000 Yuan. In medium GDP per capita regions, the hypertension prevalence decreased from 37.80% (95% CI: 35.93–39.67%) in household income of ≤4999 to 29.48% (95% CI: 28.46–30.50%) in household income of 5000–9999 Yuan, increased to 32.98% (95% CI: 32.33–33.63%) in household income of 20,000–34,999 Yuan and decreased to 32.51% (95% CI: 31.68–33.35%) in household income of ≥35,000 Yuan. The association of hypertension prevalence and household income in females from different GDP per capita areas was similar to that in the male. In high GDP per capita areas, the prevalence of hypertension decreased from 44.83% (95% CI: 43.40–46.27%) in household income of ≤4999 Yuan to 33.09% (95% CI: 32.28–33.90%) in household income of ≥35,000 Yuan. The hypertension prevalence in the low GDP per capita areas decreased from 38.11% (95% CI: 37.26–38.96%) in household income of ≤4999 to 28.26% (95% CI: 26.89–29.64%) in household income of ≥35,000 Yuan. In the medium GDP per capita areas, hypertension prevalence decreased from 25.83% (95% CI: 24.34–27.32%) in household income of ≤4999 to 22.25% (95% CI: 21.41–23.10%) in household income of 5000–9999 Yuan, and increased to 33.09% (95% CI: 32.28–33.90%) in household of ≥35,000 Yuan. Sensitivity test also demonstrated that the interation term between household income and GDP per capita was significant for both males and females (*p < 0.01*).

### Hypertension prevalence in different GDP per capita areas across other socioeconomic, lifestyle and mental health characteristics

Table 2 presents adjusted hypertension prevalence across other socioeconomic, lifestyle and mental health characteristics stratified by GDP per capita levels and gender. In general, males had higher hypertension prevalence than females. The highest prevalence of hypertension was observed in the high GDP per capita regions while the lowest in the medium GDP per capita areas. In both males and females across different GDP per capita areas, the highest hypertension prevalence was observed among those who were equal or higher than 55 years old, those who were uneducated or highest education being primary school, those unemployed, those whose MET was less than 11 h/d and being overweight. In males across different GDP per capita areas, occasional smokers and drinkers, and those who slept for more than 9 h per day had the highest hypertension prevalence. While in females, regular smokers and drinkers, and those who slept for ≤6 h per day had the highest hypertension prevalence.

**Table 2:** Adjusted Prevalence* of Hypertension (95%CI)

	Level of economic development (measured by regional GDP per capita)					
	Male			Female		
	Low	Medium	High	Low	Medium	High
Demographic characteristics						
Mean age, year						
< 55	22.60 (21.96, 23.24)	20.18 (19.42, 20.95)	32.17 (31.30, 33.04)	19.85 (19.43, 20.28)	10.74 (10.15, 11.33)	24.69 (23.68, 25.69)
≥ 55	45.01 (44.35, 45.68)	38.56 (37.6, 39.53)	49.96 (49.12, 50.79)	48.88 (48.16, 49.60)	36.26 (35.45, 37.08)	50.78 (50.04, 51.52)
Socioeconomic characteristics						
Highest education						
Uneducated and primary school	35.63 (35.14, 36.11)	31.36 (30.05, 32.67)	44.54 (43.72, 45.37)	37.17 (36.71, 37.62)	24.67 (23.92, 25.41)	42.67 (41.82, 43.53)
Middle and high school	29.14 (28.44, 29.85)	26.46 (26.00, 26.91)	36.10 (35.31, 36.89)	23.18 (22.47, 23.88)	15.16 (14.76, 15.56)	22.82 (21.84, 23.79)
College and university	28.91 (26.75, 31.06)	28.39 (27.67, 29.12)	32.26 (30.65, 33.88)	14.09 (12.11, 16.08)	12.83 (12.28, 13.39)	14.74 (13.10, 16.37)
Occupation						
Agriculture and related	29.53 (29.20, 29.86)	20.51 (19.10, 21.91)	43.22 (42.04, 44.40)	27.43 (27.07, 27.78)	11.25 (10.19, 12.32)	38.50 (36.92, 40.09)
Factory workers	23.76 (21.97, 25.54)	24.68 (23.74, 25.62)	32.53 (31.97, 33.09)	17.59 (15.84, 19.33)	10.65 (9.53, 11.77)	20.01 (19.49, 20.54)
Clerk	26.99 (26.06, 27.92)	25.08 (24.35, 25.81)	32.13 (31.36, 32.89)	18.53 (17.37, 19.68)	12.02 (11.13, 12.90)	18.80 (18.08, 19.51)
Unemployed	48.10 (46.88, 49.32)	40.99 (40.35, 41.64)	46.29 (45.26, 47.32)	43.19 (42.41, 43.96)	31.94 (31.48, 32.41)	39.57 (38.96, 40.19)
Lifestyle factors						
Physical activity (MET, h/d),%						
< 11.0	42.74 (41.91, 43.57)	37.54 (36.78, 38.30)	47.12 (45.97, 48.27)	42.51 (41.77, 43.26)	30.61 (29.98, 31.23)	42.76 (41.91, 43.62)
11.0-	31.60 (30.78, 32.42)	27.87 (26.65, 29.10)	37.16 (36.19, 38.14)	33.74 (32.99, 34.49)	23.14 (22.37, 23.91)	35.64 (34.58, 36.70)
Men 19.0-,Women 17.0	26.92 (25.96, 27.87)	21.32 (20.65, 21.99)	37.23 (36.16, 38.30)	23.25 (22.61, 23.90)	13.87 (12.89, 14.86)	29.58 (27.95, 31.21)
Men 32.5-,Women 28.5-	23.55 (22.69, 24.41)	20.46 (19.18, 21.73)	35.87 (34.76, 36.97)	22.41 (21.49, 23.34)	9.19 (8.07, 10.31)	27.5 (26.12, 28.89)
BMI (kg/m2), %						
< 18.5	22.50 (21.47, 23.54)	17.93 (16.31, 19.55)	33.32 (31.14, 35.51)	21.56 (20.50, 22.63)	13.40 (12.08, 14.72)	27.64 (25.86, 29.41)
18.5-	26.45 (25.95, 26.95)	20.91 (20.12, 21.70)	34.09 (33.25, 34.93)	23.52 (23.07, 23.98)	13.96 (13.34, 14.58)	27.60 (26.61, 28.60)
24.0-	38.49 (37.54, 39.44)	35.68 (35.00, 36.35)	45.74 (44.99, 46.50)	37.24 (36.46, 38.03)	24.51 (23.75, 25.27)	38.93 (38.00, 39.86)
> 28.0	50.73 (49.17, 52.28)	52.28 (49.84, 49.84)	55.01 (55.01, 55.01)	50.05 (48.71, 51.40)	39.89 (38.79, 41.00)	52.70 (51.53, 53.86)
Smoking						
Never	34.8 (33.67, 35.93)	30.98 (30.98, 31.73)	42.41 (41.25, 43.57)	30.94 (30.54, 31.34)	19.88 (19.44, 20.32)	33.89 (33.26, 34.53)
Occasional	36.89 (36.05, 37.74)	33.02 (32.30, 33.75)	44.35 (43.39, 45.31)	32.56 (31.17, 31.17)	27.34 (25.94, 28.73)	40.13 (37.73, 42.54)
Regular	29.46 (28.90, 30.02)	25.73 (24.98, 26.48)	37.27 (36.54, 38.01)	32.94 (31.36, 34.51)	27.69 (26.08, 29.30)	45.21 (42.14, 48.28)
Alcohol						
Never	34.05 (32.68, 35.42)	30.42 (29.49, 31.35)	42.72 (41.41, 44.03)	30.76 (30.35, 31.18)	19.33 (18.79, 19.87)	34.19 (33.43, 34.94)
Occasional	36.73 (35.75, 35.75)	31.54 (30.15, 32.92)	43.57 (42.51, 44.64)	30.90 (29.46, 32.34)	26.39 (24.96, 27.83)	39.34 (34.95, 43.74)
Regular	29.39 (28.74, 30.03)	25.20 (24.02, 26.38)	37.49 (36.65, 38.32)	31.60 (29.87, 33.34)	27.54 (25.89, 29.19)	42.84 (38.00, 47.68)
Sleep duration/day,%						
≤ 6 h	32.32 (31.62, 33.03)	32.89 (32.20, 33.59)	41.42 (40.44, 42.40)	35.10 (34.36, 35.84)	25.61 (24.95, 26.28)	38.98 (38.14, 39.82)
> 6 h, ≤9 h	31.27 (30.77, 31.76)	27.60 (27.06, 28.13)	38.53 (37.96, 39.11)	29.77 (29.32, 30.21)	18.70 (18.18, 19.21)	32.53 (31.84, 33.22)
> 9 h	35.10 (34.26, 35.94)	35.38 (33.78, 36.98)	44.49 (43.20, 45.78)	30.17 (29.31, 31.04)	23.93 (22.00, 25.86)	39.75 (38.35, 41.15)
Depression	31.76 (30.29, 33.22)	30.77 (29.25, 32.29)	35.66 (34.31, 37.01)	30.98 (29.14, 32.83)	18.08 (15.74, 20.43)	30.76 (28.13, 33.39)
Anxiety	25.79 (24.05, 27.53)	17.65 (17.65, 17.65)	29.41 (29.41, 29.41)	25.50 (23.32, 27.67)	20.91 (17.70, 24.12)	24.34 (21.78, 26.91)

* Adjusted for the other covariates except the examined variable. The total covariates included age the time of study, occupation, education, MET, BMI, alcohol, smoke, sleeping time, anxiety, depression, household income and household size

## Discussion

Several important findings were illustrated in the present study. First, hypertension prevalence was influenced by regional economic status. The prevalence of hypertension was the lowest in the medium GDP per capita areas compared to that in the low and high GDP per capita areas. Second, there was an inverse relationship between hypertension and household income in the low and high GDP per capita areas, and an U-shaped association between hypertension and household income in the medium GDP per capita areas. Third, higher hypertension prevalence was observed in males than females across all GDP per capita regions, and the degree of the negative correlation between household income and hypertension appeared to be higher in females in the low and high GDP per capita areas.

The present analysis used the baseline data from a large population-based study in China, which covered diverse geographic and socioeconomic areas, and used standardized techniques in measuring blood pressure. Though the response rate was relatively low, the large sample size and diverse geographic coverage ensured the credibility of our findings. There are few articles examining hypertension prevalence in economically diverse regions in China [26, 27]. The findings of the present study provide insight into the influence of regional economic conditions on hypertension prevalence and the correlations between sex, household income, and hypertension prevalence under different GDP per capita regions. Admittedly, there were several limitations of the present study. First, blood pressure was measured on one occasion whereas ideally two or more measurements should be taken on separate occasions. Diagnosis based on serial measurements on one occasion might affect the prevalence estimation. Second, we used the midpoint of categorical income variable in the present study to represent the income distribution, which was a proxy and may influence the accuracy of income distribution. Third, although we have adjusted for many hypertension risk factors in the model, residual confounders might still exist. Meanwhile, the cross-sectional nature of the study is not sufficiently strong to establish a causal relationship of socioeconomic status and hypertension. Fourth, the data of this study was collected about 10 years age, which cannot present the current situation in China because of its fast economic development. However, we believed our findings could provide information for other middle income and developing countries. Lastly, the ten study areas were not selected as representatives of China, but the large sample size and diverse geographic coverage ensured the validity of our findings.

In the present study, the prevalence of hypertension in China was the lowest in the medium GDP per capita areas but highest in high GDP per capita areas. From a global perspective, the highest hypertension prevalence has shifted from high-income countries to low- and middle-income countries, where hypertension prevalence is the highest in low-income countries [2]. Better economic development is generally conducive to better health [11], as is verified by the declined hypertension prevalence in high-income countries [2]. However, socioeconomic status is found to be positively associated with hypertension in undeveloped or developing countries, which is often accompanied by obesity, and high salt and alcohol intake among people in a high socio-economic class [13, 28]. Our data also proved that overweight and obesity prevalence was the highest in high GDP per capita areas. China, as a middle-income country, has undergone rapid urbanization in the past three decades [5], which brings regional disparity in economy and social development [6]. Transitioning from low to high, the medium GDP per capita regions undergo rapid social and economic development [6]. We propose that differences in the accessibility of healthcare facilities, environmental factors and dietary habits among different GDP per capita areas might explain the low hypertension prevalence in the medium GDP per capita areas. Compared with low GDP per capita areas, healthcare facilities are generally better in medium GDP per capita areas [28], providing more advanced healthcare and hypertension management. As compared to high GDP per capita regions, environmental risk factors for hypertension, such as noise and air pollution [29], may be less prominent in the medium GDP per capita areas, since economic growth is frequently accompanied with damage to the natural environment [30]. Furthermore, as the regional economy develops, people generally tend to adopt healthier lifestyles, such as a healthier diet and more physical activities, which may also contribute to a lower hypertension prevalence [2]. Workplace stress and pressure in life may be more prevailing in high GDP per capita areas, constituting a higher risk of hypertension [31]. Depression associated with stress and pressure in high GDP per capita areas may also be a risk factor for hypertension [32].

In both sexes, there existed an inverse association between household income and hypertension in the low and high GDP per capita areas and a U-shaped association between household income and hypertension in the medium GDP per capita areas. As pointed out in previous studies, socioeconomic status is negatively associated with hypertension in the developed countries [13, 33, 34], which is consistent with our observations in the low and high GDP per capita areas. Residents with higher income may be able to afford a healthier lifestyle, including a healthier diet and more physical acitivities, and benefit from accessiblity to resources such as better and more advanced healthcare facilities. All of such efforts may lower the risks of hypertension, and may be related to the lower hypertension prevalence observed in the higher income groups from both low and high GDP per capita areas. Our finding is supported by a study conducted in Jamaica which found that hypertension prevalence was elevated in low and high income groups [35]. In the medium GDP per capita regions, rapid transitioning and developement have brought about socioeconomic disparity [6]. Lower-income residents may be exposed to more risk factors for hypertension [2, 8], including higher sodium intake, smoking, drinking, and undernutrition in early life [36]. While in the higher-income group, workplace pressure and sedentary lifestyle are more prevailing risk factors for hypertension [37]. Different income groups in the medium GDP per capita areas may be affected by different risk factors, thus forming the U-shaped association.

Prevalence of hypertension in males was generally higher than that in females, and the negative association was stronger in females. Previous studies have suggested that males are at higher risk for hypertension than females [38–40]. Research exploring sex differences in the cardiovascular system suggested that males are at higher risk for higher blood pressure than age-matched premenopausal women via androgen-mediated effect on Renin-angiotensin system [40, 41]. In addition, males tend to consume more fat and sugar than females, and face intenser workplace stress, thus have an increased risk for hypertension [42]. Overall, there was an inverse association between household income and hypertension prevalence, and the association is stronger in females than in males. We speculate that social factors may also be related to this finding. As income increases, females tend to be more attentive than males to personal health management [38, 43], make better use of health facilities and adopt healithier lifestyles [44] which all may lower hypertension risks. Due to the perception of beauty, affluent Chinese women are more attentive to appearance and value slenderness [45], thus more aware of a healthy diet and engage in more physical activity. Therefore, physiological and social factors all contribute to sex differences in hypertension prevalence.

## Conclusions

In the present study, the prevalence of hypertension was lower in the medium GDP per capita areas as compared to that in the low and high GDP per capita areas. The different shapes of associations between household income and hypertension prevalence were observed in different GDP per capita areas. There were strong gender differences in terms of both trend and strength of the association between household income and hypertension prevalence. The present study underlined the importance of regional economic development, household income and sex differences in relation to hypertension prevalence. Interventions for hypertension prevention and management should take into consideration the influence of gender difference and socioeconomic disparities at both macro and micro levels, in addition to a person-centered approach.

## Supplementary information

**Additional file 1.** Supplemental tables and figures for the manuscript.

## Data Availability

The datasets used and/or analysed during the current study are available from the corresponding author on reasonable request.

## Abbreviations

CKB: China Kadoorie Biobank
MET: Metabolic Equivalent Task
BMI: Body Mass Index
CIDI-SF: Composite International Diagnostic Inventory - Short Form
