# Supplementary material for: Regional economic development, household income, gender and hypertension: evidence from half a million Chinese
Source: BMC Public Health. 2020 Jun 10;20:901. doi: 10.1186/s12889-020-09002-y (PMC7288507; doi:10.1186/s12889-020-09002-y)
Supplement: Supplementary file 1 — Additional file 1. Supplemental tables and figures for the manuscript. [file 12889_2020_9002_MOESM1_ESM.docx]

**Appendix**

***Appendix 1*** ***Conceptual framework for the interplay of economic development, income and hypertension prevalence, using evidence from previous literature.***

***
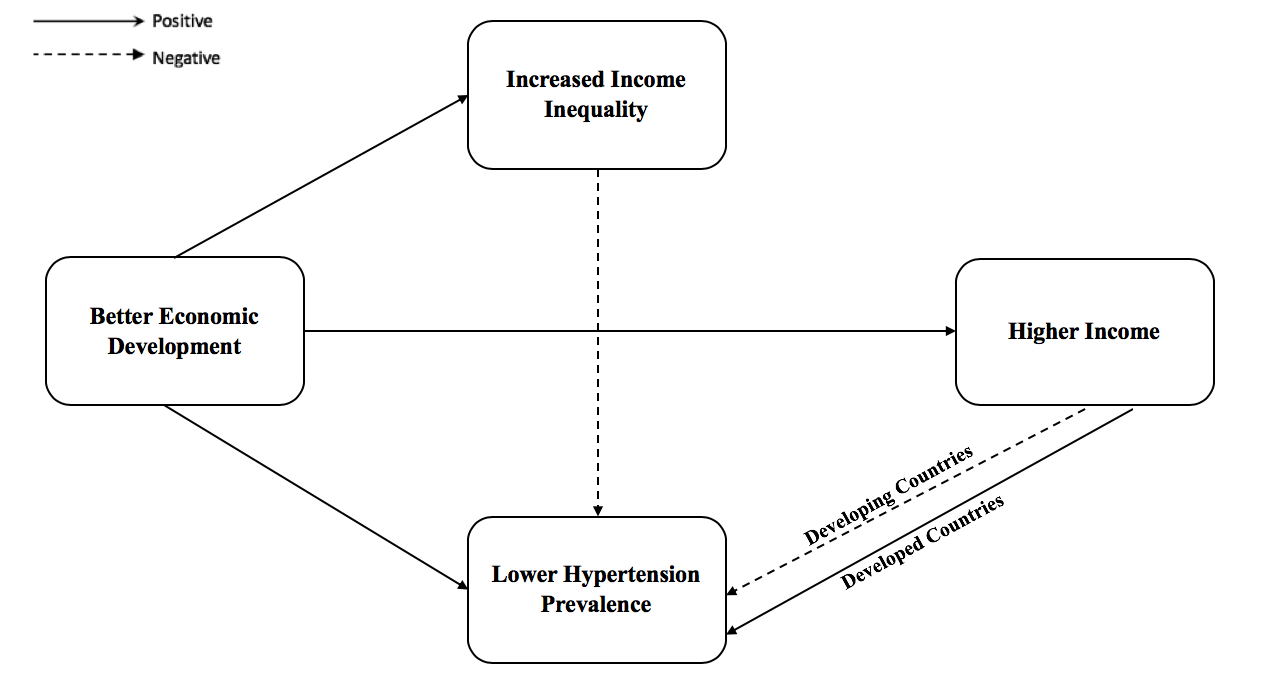
***

***Appendix 2 GDP per capita for different regions and study region map***

| Study Sites | GDP per capita (2007) | Type | Economic Development |
| --- | --- | --- | --- |
| Qingdao, Shandong | 44964 | Urban | High |
| Harbin, Heilongjiang | 24306 | Urban | Medium |
| Haikou, Hainan | 22258 | Urban | Medium |
| Suzhou, Jiangsu | 69151 | Urban | High |
| Liuzhou, Guangxi | 20268 | Urban | Medium |
| Pengzhou, Chengdu, Sichuan. | 14028 | Rural | Low |
| Maijixiang, Tianshui, Gansu. | 5550 | Rural | Low |
| Huixian, Henan | 14950 | Rural | Low |
| Tongxiang, Zhejiang. | 41000 | Rural | High |
| Liuyang, Hunan | 19197 | Rural | Low |

***Appendix 3 Univariate analyses results for covariates and hypertension.***

| Covariates | Odds Ratio (95%CI) |
| --- | --- |
| Age | 2.13 (2.12, 2.15) |
| Occupation |  |
| Agriculture and related | ref |
| Factory workers | 0.79 (0.77, 0.8) |
| Clerk | 0.69 (0.68, 0.7) |
| Unemployed | 1.49 (1.47, 1.51) |
| Highest Education |  |
| Uneducated and primary school | ref |
| Middle and high school | 0.55 (0.54, 0.56) |
| College and university | 0.48 (0.47, 0.49) |
| MET (h/d) | 0.99 (0.99, 0.99) |
| BMI (kg/m2) | 1.68 (1.66, 1.69) |
| Alcohol |  |
| Never | ref |
| Occasional | 0.76 (0.75, 0.77) |
| Regular | 1.04 (1.02, 1.06) |
| Smoking |  |
| Never | ref |
| Occasional | 1.36 (1.34, 1.39) |
| Regular | 1.07 (1.05, 1.08) |
| Sleeping Time (h) | 0.98 (0.98, 0.98) |
| Household income |  |
| ≤4,999 yuan | ref |
| 5,000-9,999 yuan | 0.67 (0.65, 0.68) |
| 10,000-19,999 yuan | 0.68 (0.67, 0.69) |
| 20,000-34,999 yuan | 0.70 (0.68, 0.71) |
| ≥35,000 yuan | 0.69 (0.67, 0.71) |
| Household Size |  |
| ≤2 | ref |
| 3 | 0.44 (0.43, 0.45) |
| 4 | 0.52 (0.51, 0.53) |
| ≥5 | 0.76 (0.75, 0.78) |
| Depression |  |
| No | ref |
| Yes | 0.86 (0.80, 0.93) |
| Anxiety |  |
| No | ref |
| Yes | 0.72 (0.63, 0.82) |

***Appendix 4 Adjusted Mean Systolic and Diastolic Blood Pressure Across Different GDP Per Capita Areas***


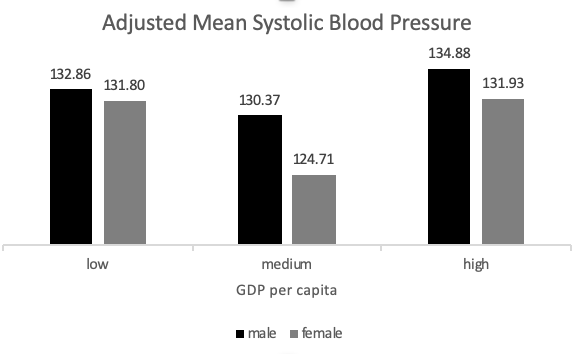

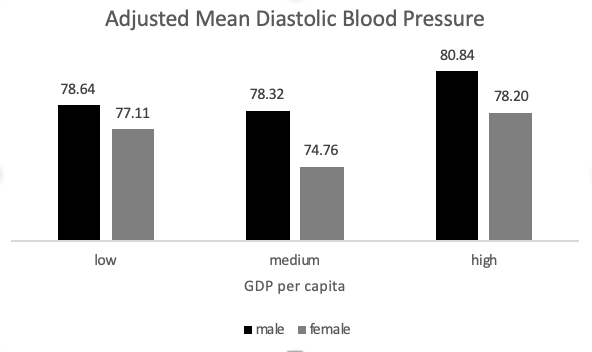


*Adjusted for age, occupation, education, MET, BMI, alcohol, smoke, sleeping time, anxiety, depression, household income and household size.*

***Appendix.5 Adjusted* Prevalence of Overweight and Obesity Across Different GDP Per Capita Areas***

|  | GDP per capita | | |
| --- | --- | --- | --- |
|  | Low | Medium | High |
| Overweight  (24.0 <BMI≤ 28.0 kg/m^2^) | 29.44% (30.54, 31.22) | 29.37% (28.67, 30.06) | 33.58% (32.97, 34.19) |
| Obesity  (BMI>28.0 kg/m^2^) | 8.85% (8.64, 9.06) | 8.77% (8.40, 9.14) | 12.28% (11.94, 12.61) |

**Adjusted for age occupation, education, alcohol, smoke, sleeping time, anxiety, depression, household income and household size.*
